# ebTrack: an environmental bioinformatics system built upon ArrayTrack™

**DOI:** 10.1186/1753-6561-3-s2-s5

**Published:** 2009-03-10

**Authors:** Minjun Chen, Jackson Martin, Hong Fang, Sastry Isukapalli, Panos G Georgopoulos, William J Welsh, Weida Tong

**Affiliations:** 1 Department of Environmental and Occupational Medicine, UMDNJ-RWJMS, 675 Hoes Lane, Piscataway, NJ 08854, USA; 2 Department of Pharmacology, UMDNJ-RWJMS, 675 Hoes Lane, Piscataway, NJ 08854, USA; 3 Z-Tech Corporation, an ICF International Company at National Center for Toxicological Research (NCTR), US Food and Drug Administration, 3900 NCTR Road, HFT 230, Jefferson, AR 72079, USA

## Abstract

ebTrack is being developed as an integrated bioinformatics system for environmental research and analysis by addressing the issues of integration, curation, management, first level analysis and interpretation of environmental and toxicological data from diverse sources. It is based on enhancements to the US FDA developed ArrayTrack™ system through additional analysis modules for gene expression data as well as through incorporation and linkages to modules for analysis of proteomic and metabonomic datasets that include tandem mass spectra. ebTrack uses a client-server architecture with the free and open source PostgreSQL as its database engine, and java tools for user interface, analysis, visualization, and web-based deployment. Several predictive tools that are critical for environmental health research are currently supported in ebTrack, including Significance Analysis of Microarray (SAM). Furthermore, new tools are under continuous integration, and interfaces to environmental health risk analysis tools are being developed in order to make ebTrack widely usable. These health risk analysis tools include the Modeling ENvironment for TOtal Risk studies (MENTOR) for source-to-dose exposure modeling and the DOse Response Information ANalysis system (DORIAN) for health outcome modeling. The design of ebTrack is presented in detail and steps involved in its application are summarized through an illustrative application.

## Background

Toxicogenomics [1,2], with its emphasis on analysis of gene expression, metabolic pathways, and regulatory networks, has revolutionized the field of toxicology. Toxicogenomics combines the technologies of genomics, proteomics, and metabonomics (collectively called "omics") to identify and characterize mechanisms of action of known and suspected toxicants as well as to determine predictive biomarkers (or expression signatures) for supporting health risk assessments. Therefore, an integrated bioinformatics system that enables consistent analysis of collective "omics" data sets and meta data is crucial for toxicogenomics research. Such a system must implement the following three critical features in order to facilitate the successful analysis and interpretation of toxicogenomics studies:

(1) *Centralized data management and annotation *of data from various types of "omics" experiments and multiple steps within each experiment. This facilitates a common starting point for comparative application of independent tools and analysis methods by different researchers;

(2) *Provision for rapid data analysis *via an intuitive user interface. This facilitates easy application of standard tools for quickly searching, filtering, applying mathematical and statistical operations, and graphically visualizing various "omics" data sets; and

(3) *Provision for informed data interpretation *through interfaces to relevant knowledge bases of gene annotation, protein function and pathways. This facilitates efficient and effective data interpretation, and allows for rapid re-examination and analysis of data sets utilizing updated toxicogenomic knowledge (e.g. updated gene annotation, metabolic pathway information, etc.)

The following section describes the design of ebTrack, which is being developed as an integrative bioinformatics system that includes the aforementioned features in relation to applications of toxicogenomics in environmental health research. Specifically, ebTrack is being developed as an enhancement to the US FDA's ArrayTrack™ system [3,4], which facilitates the data management and analysis of gene expression microarray data. The main objective in the development of ebTrack is to provide a one-stop solution for environmental research by addressing the issues of integration, curation, management, analysis and interpretation of environmental and toxicological data from diverse types of experiments encountered in environmental health research.

## Methods

ArrayTrack™ has been developed at the FDA's National Center for Toxicological Research (NCTR) over the past seven years and is a comprehensive microarray data management, analysis and interpretation system. It is currently being used in the FDA as a review tool for Voluntary Genomics Data Submission (VGDS). However, multiple factors necessitate the development of a new system based on ArrayTrack™: (a) it is based on a client-server architecture involving expensive database software (Oracle; ); (b) it is limited to the management and analysis of data from DNA microarray experiments for humans, mouse and rat; and (c) it is not a public repository and, thus, still requires significant scale up in order to accommodate data from diverse laboratories involved in environmental health research; and (d) it was originally developed by the US FDA for regulatory purposes, and thus incorporates a set of well-established tools that comprise only a small subset of the tools required to address the broad scope and variation in data sets encountered in environmental health research. Therefore, ebTrack is being developed as an enhancement to ArrayTrack™ in order to provide a significantly wider set of tools and functionality necessary for environmental bioinformatics and quantitative risk assessment.

The design of ebTrack includes individual modules for management, analysis and interpretation of data from single or multiple experiments that span multiple biological scales (transcriptomics, proteomics, and metabonomics); these modules are designed for use independently or in combination, as desired by the end user. The modular structure allows for a gradual development, implementation, and application of ebTrack as a user-oriented system for integrated systems toxicology studies. Specifically, users of ebTrack will be able to select an analysis method, apply it to the stored "omics" data, and link the analysis results to gene, protein and pathway information for further data interpretation. Likewise, they will be able to analyze and visualize various "omics" data sets in conjunction with traditional toxicological data for enhanced interpretation of all available data through systems biology.

ebTrack is being developed as a client-server system, using the powerful, free, open source PostgreSQL database engine, and Java tools for user interface, analysis, visualization, and web-based deployment. JDBC (Java Database Connectivity) is used for querying the database, since it facilitates cross-platform deployment as well as integration with other databases, including Oracle. Furthermore, the use of Java tools in ebTrack allow for direct integration with other environmental tools such as the Modeling ENvironment for TOtal Risk studies (MENTOR [5,6]), which provides an open library of computational tools for exposure and dose modeling, and the DOse Response INformation ANalysis system (DORIAN;[7]), which is under development as a toolbox for modeling processes in the sequence from dose to adverse health outcome. Both MENTOR and DORIAN are based on Matlab, which provides direct interfaces for invoking functions written in other languages such as C, C++, FORTRAN, Java, and Perl.

The implementation of ebTrack in the context of a typical deployment involves the following modules: (a) the database module stores experiment information (e.g. gene expression microarray data or tandem mass spectrometry data) in accordance with standard protocols; (b) the analysis tools module provides tools for analysis, visualization, and knowledge discovery; and (c) the functional data module provides relevant information for data interpretation. The overall architecture and design of ebTrack is similar to that of ArrayTrack™, and the design is based on a full integration of the above modules for facilitating consistent analysis of diverse toxicogenomic data sets for environmental health risk analysis. These three major modules are described in the following:

### (1) Database Module

This supports toxicogenomics data in various standard guidelines and formats such as the MIAME (Minimum Information About a Microarray Experiment [8]) and MAGE-ML standards (Microarray Gene Expression Markup Language [9]). This module is designed to accommodate a wide set of experimental data relevant to environmental toxicology and to facilitate easy exchange of data with other public repository such as ArrayExpress, Gene Expression Omnibus (GEO), ArrayTrack™, and Chemical Effects in Biological Systems (CEBS) [3,8,10,11].

### (2) Analysis Tools Module

In order to meet the variety of analysis needs for environmental health research, ebTrack is designed as an open architecture to incorporate diverse analysis tools from different sources. In addition to the analysis tools supported by ArrayTrack™, this module contains interfaces to public bioinformatics tools and resources such as R/Bioconductor [12]. As an example, the Significance Analysis of Microarray (SAM [13]), which is a widely used method for gene expression data analysis was implemented as an ebTrack module through a customized interface for running SAM in ebTrack. This design also allows straight-forward incorporation of other analysis tools in Bioconductor into ebTrack while providing a consistent user interface. This module also contains tools for exporting the data in ebTrack to an R environment for analysis utilizing user-selected custom tools in R. Furthermore, interfaces are being developed to use these tools with the MENTOR and DORIAN systems that provide modules for mechanistically modeling various processes in the source-to-dose-to-outcome continuum (see, e.g., [7]).

### (3) Functional Data Module

Large amounts of annotation data in public domain for different organisms (e.g. human, rat, mouse, canine and zebrafish) is being compiled through local mirroring of publicly available data; the data is stored in standard formats in order to facilitate rapid analysis, and easy interoperability with other tools and databases. ebTrack also contains provisions for connecting to various proprietary databases for interpreting various "omics" studies. An Environmental Bioinformatics Knowledge Base (ebKB; ), a compendium of computational tools, databases, and literature information, is being developed as part of this effort by the environmental bioinformatics and Computational Toxicology Center (ebCTC; ) to support enhanced interpretation of various toxicological and biological data sets in ebTrack.

**Figure 1:**
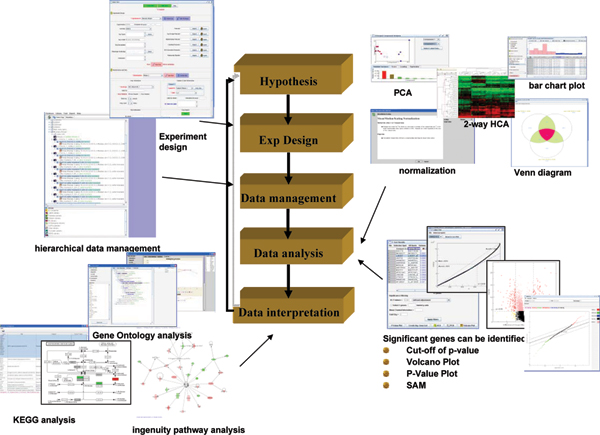
Steps involved in the application of ebTrack for microarray data analysis

A workflow involving the important steps for the analysis of microarray data using ebTrack is presented in Figure 1. This approach has been applied in a case study focusing on gene expression profiles of mouse skin after a single high dose of Sulfur Mustard (SM) applied topically. SM is a chemical warfare agent that can penetrate human skin causing extensive blistering at the dermal-epidermal junction after a latency period of several hours. Although toxic effects of SM have been well characterized, the precise mechanisms responsible for SM-induced skin injury are still unknown. In this study, the effects of SM-treatment on mouse skin were examined at multiple time points to characterize the extended time response. This study was also used to evaluate the efficacy of candidates for inhibiting the adverse effects of SM. The study was done by (a) identifying a list of differentially expressed genes using the volcano plot (p < 0.05 and fold-change > 1.5) and SAM algorithm, (b) mapping those genes to KEGG (Kyoto Encyclopedia of Genes and Genomes [14]) and Ingenuity Pathway Analysis (; see, e.g. [15]) and (c) determining significant pathways using a Fisher Exact test. The results indicated that cytokine-cytokine receptor interaction, cell adhesion molecules and hematopoietic cell lineage are common significant pathways in the mouse skin treated with SM. Details on the study are available in Gerecke et al. (2008; unpublished manuscript, revisions to the manuscript submitted).

## Discussion

A comprehensive bioinformatics system, ebTrack, is being developed focusing on supporting toxicogenomics-driven environmental health research. This process is critical in assessing disease susceptibility with respect to specific environmental agents, and requires data/information integration on the effects of xenobiotics across multiple biological levels (genes, proteins, metabolites) to determine the corresponding impact on bionetwork (signaling, regulatory, metabolic) dynamics. It is being built upon the US FDA's ArrayTrack™ system in a modular manner with an open architecture to facilitate rapid development and integration of modules that handle data from new types of experiments.

The approach being followed in the development of ebTrack, in conjunction with related computational tools, is targeted towards supporting the development of generalized, integrated, physiologically based models for the "coupled" toxicokinetics and toxicodynamics of contaminants of concern and their mixtures [16,17], by accounting for variability in the signaling, gene-regulatory and metabolic processes involving environmental contaminants, which in turn affect the distribution of responses (health impact) within a human population.

Therefore, ebTrack is designed to handle, at a minimum, the following types of data: gene expression data from various platforms of DNA microarray (e.g. Affymetrix, Agilent, custom arrays); proteomics data (e.g. Ciphergen ProteinChips, 2D-gel electrophoresis, mass spectrometry); metabonomics data (e.g. mass spectrometry and NMR); chemical structure information and *in vitro/in vivo *toxicological data. In addition to the functionality and tools of ArrayTrack™, a broad array of data analysis tools are being implemented in ebTrack and open interfaces are being implemented in order to facilitate its wider use.

## Disclaimer

The views presented in this article do not necessarily reflect those of the US Food and Drug Administration, the US Environmental Protection Agency, or the National Institute for Environmental Health Sciences

## Competing interests

We declare that we have no conflict of interest

## Authors' contributions

MC developed the system, and wrote manuscript draft. JM helped developing the database system. SI, PGG and WJW contributed to design the framework of the sytem, and helped wrting the manuscipt. WT and HF helped to design and test the system, and writing the manuscript. All authors participated in preparation of the manuscript, and approved its final form.
